# Pathogenic variants in *BRCA1* and *BRCA2* genes associated with female breast and ovarian cancer in the Mexican population

**DOI:** 10.25122/jml-2024-0213

**Published:** 2025-01

**Authors:** Flor Daniela Alday-Montañez, Daniel Dickens-Terrazas, Gloria Erika Mejia-Carmona, Elisa Robles-Escajeda, Robert Arthur Kirken, Alfonso Enrique Bencomo-Alvarez, Victor Josue Carrasco-Urrutia, Naun Lobo-Galo, Luis Fernando Plenge-Tellechea, Ángel Gabriel Diaz-Sanchez, Alejandro Martínez-Martínez

**Affiliations:** 1 Department of Chemical-Biological Sciences, Autonomous University of Ciudad Juarez, Ciudad Juarez, Chihuahua, Mexico; 2 IMSS, Zone Hospital Number 6, Ciudad Juarez, Chihuahua, Mexico; 3 Department of Biological Sciences, The Border Biomedical Research Center, The Cellular Characterization and Biorepository Core Facility, The University of Texas, El Paso, Texas, USA; 4 Department of Host-Microbe Interactions, St. Jude Children’s Research Hospital, Memphis, TN, USA; 5 Endofem, Ciudad Juarez, Chihuahua, Mexico

**Keywords:** founder effect, Mexican population, breast cancer, ovarian cancer, genetic variation, BC, Breast cancer, OC, ovarian cancer, HBOC, hereditary breast and ovarian cancer syndrome (HBOC), *BRCA1*, Breast Cancer Susceptibility Gene 1, *BRCA2*, Breast Cancer Susceptibility Gene 2, HRR, Homologous Recombination Repair pathway, PALB2, Partner and Localizer of *BRCA2*, RAD-51, DNA repair protein RAD-51 homolog 1, BARD1, *BRCA1*-associated RING domain protein-1, TNBC, triple-negative breast cancer, CR, mutation-specific cluster regions, SNVs, Single Nucleotide variants, TCGA, The Cancer Genome Atlas, MLPA, multiplex ligation-dependent probe amplification

## Abstract

Breast and ovarian cancers are significant global health challenges, with inherited variations in breast cancer gene 1 (*BRCA1*) and breast cancer gene 2 (*BRCA2*) substantially increasing the risk, aggressiveness, and early onset of these diseases. This work aimed to examine pathogenic variants (PVs) in *BRCA1* and *BRCA2* databases that include Mexican populations. A systematic review of literature and data mining spanning from 2002 to 2023 was conducted. Articles published in journals indexed in SCImago quartiles Q1 to Q4 were screened. Databases were paired, standardized, and enriched with data from reputable global platforms: Genome Data Viewer, dbSNP, ClinVar, gnomAD browser, Breast Cancer Information Core (BIC), ClinGen, Varsome, Human Genome Variation Society (HGVS), Bioproject, Ensembl, Gene NIH NCIB, UniProt, and BRCA Exchange. Outcomes included data from 9,026 Mexican genotypes, identifying 657 PVs. Genetic mapping revealed certain exons, notably exon 10 of *BRCA1* and exon 11 of *BRCA2*, as highly mutagenic hot spots. The most frequent alteration was a large deletion in *BRCA1* (ex9-12del), associated with a founder effect originating from a common Mexican ancestor. Finally, we constructed a genetic map containing all the single nucleotide variants (SNVs) and large rearrangements presented in the analyzed databases.

## INTRODUCTION

Breast and ovarian cancer syndrome (HBOC), linked to inherited variations, presents significant challenges in women's health worldwide [1]. Breast cancer (BC) is particularly pervasive, accounting for the highest number of new cancer cases among women globally. In 2022 alone, 2.3 million women were diagnosed with BC, leading to 666,103 deaths and making it the main cause of cancer-related mortality in women worldwide. Conversely, ovarian cancer (OC) ranks as the sixth most common gynecologic cancer but carries the highest mortality rate among these cancers [2]. Despite its lower prevalence compared to BC, OC is three times more lethal, underscoring the urgent need for effective screening and treatment strategies [3].

There are disparities in breast cancer incidence and tumor type based on ethnicity and race. US-born Hispanics with triple-negative breast cancer (TNBC) have a poorer prognosis compared to other racial groups, and both they and Mexico-born Hispanics experience worse outcomes in luminal breast cancer compared to non-Hispanic whites. These differences highlight the complexity of disparities between Hispanic populations, particularly Mexican and Caucasian populations [4]. The elevated mortality rates associated with BC and OC are particularly exacerbated in developing regions, where limited access to healthcare, early detection, and treatment is limited [5-7].

Dysfunction in breast cancer susceptibility gene 1 (*BRCA1*) and *BRCA2* is strongly associated with genomic instability, as these genes encode proteins essential for DNA repair and cell cycle regulation [8]. Consequently, they are key biomarkers linked to a high risk of BC and OC [9]. *BRCA1* and *BRCA2* are associated with nearly 40% of hereditary BC cases, making their pathogenic variants (PVs) the most common genetic contributors to hereditary breast and ovarian cancer syndrome (HBOC) due to their high penetrance [10,11]. Beyond BC, *BRCA1* and *BRCA2* also play roles in other types of cancer, such as melanoma, pancreatic, prostate, biliary tract, esophageal cancer, and gastric cancer [12-14].

*BRCA1* and *BRCA2* play critical roles as tumor suppressor genes, indispensable for DNA repair [15]. Additionally, they are involved in key cellular processes such as chromatin remodeling, transcription, and cell cycle regulation. Their primary function lies in the homologous recombination repair (HRR) pathway of double-strand DNA, one of the most important pathways for maintaining genomic integrity and preventing cancer formation [16].

*BRCA1* is located on chromosome 17q21, consists of 24 exons, and encodes a protein comprising 1863 amino acids [15]. Known for its multifunctionality, *BRCA1* interacts with numerous other proteins, with experimental studies identifying 52 interactions documented in String by EXPASY [17].

On the other hand, the *BRCA2* gene is situated on chromosome 13q12-13 and comprises 27 exons, encoding a protein of 3418 amino acids [12]. This gene serves multiple functions, acting as a transcriptional coregulator and a critical tumor suppressor gene responsible for maintaining genomic integrity. Furthermore, the *BRCA2* protein plays an essential role in the HRR pathway, interacting with RAD-51 [18].

By 2023, over 3,400 PVs have been identified in *BRCA1* (1,600) and *BRCA2* (1,800). PVs in *BRCA1* raise the risk of BC by 65% and OC by 39%, while *BRCA2* PVs increase these risks by 45% and 11%, respectively. *BRCA1* PVs predispose to invasive ductal BC (~75%) or atypical medullary BC (~10%), with TNBC occurring in 66-100% of cases. *BRCA2* PVs are linked to invasive ductal BC (~75%), atypical medullary BC (<10%), and TNBC in 14-35% of cases [12].

Although PVs have been reported across all exons of the *BRCA1* and *BRCA2* genes, variation hotspots highly associated with the development of breast cancer have been identified. In the *BRCA1* gene, three highly mutated domains are significant:

1) Exons 2 to 7, which encode the cysteine-rich zinc-binding RING domain responsible for binding to *BRCA1*-associated RING domain protein 1 (BARD1). Together, they form a heterodimer that facilitates homologous recombination repair (HRR) by functioning as an E3 ubiquitin ligase [19,20].

2) Exons 11-13, which contain domains for binding to a variety of proteins. Additionally, exon 11 has been shown to be directly involved in nuclear localization signaling, making it vital for cell cycle control and DNA damage repair [19,21].

3) Exons 16-24, which encode the BRCT domain (carboxy-terminal domain of the *BRCA1* protein), which is a phosphoprotein-binding domain specific for phosphorylated proteins such as ATM/ATR kinases and many others [19,20].

In the *BRCA2* gene, several domains have also been identified as susceptible to pathogenic variations, as confirmed by analyses using Expasy and UniProt:

1) Exons 1 to 2, which encode the partner and localizer of the *BRCA2* (PALB2) binding domain.

2) Exon 11, the largest one encoding this protein, because it contains domains for binding to proteins such as NPM1, RAD-51, and POLH, which are required for DNA polymerization activity.

3) Exon 18, which contains motifs corresponding to nuclear localization signaling.

Identifying PVs in *BRCA1* and *BRCA2* carries significant clinical implications, enabling personalized therapy [22]. Mutation-specific cluster regions (CR) in both genes have been identified for breast (BCCR) and ovarian (OCCR) cancer, aiding in the monitoring of patients with PVs in *BRCA1* and *BRCA2* [23].

Genomic screening programs are urgently needed for widespread public BRCA PVs screening, especially targeting high-risk individuals using more population-specific variants [9]. In Latin America, young women have nearly double the incidence of cases compared to developed countries, with a higher prevalence of TNBC, suggesting a high-risk population for BRCA variation. However, access to genetic cancer risk assessment and testing is limited and lacks population-specific data on PVs [24]. The only genetic test tailored for the Latin American population to detect BRCA mutations is HISPANEL, which covers 114 BRCA PVs based on data from US Hispanics but lacks a specific focus on Mexico [25].

Given the distinctive genetic profile of the Mexican population, characterized by a notable incidence of BRCAs PVs, early onset, and high prevalence of TNBC (~23%), there is a compelling need to develop affordable and accessible screening for detecting BRCA PVs-specific for this population [9].

This research compiles all reported germline PVs in *BRCA1* and *BRCA2* within the Mexican population, aiming to lay the groundwork for improving the detection of these PVs.

## MATERIAL AND METHODS

**Figure 1:**
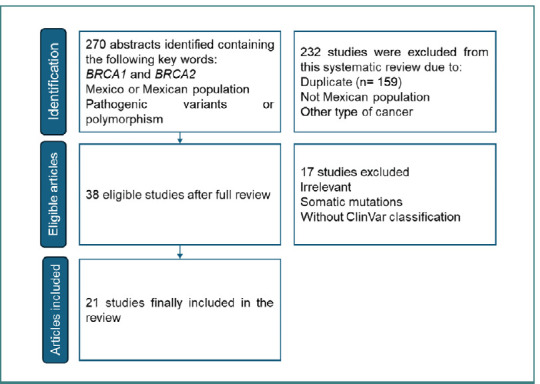
PRISMA flow chart. Mining data was extracted using the PRISMA methodology

A systematic review was conducted in accordance with the Preferred Reporting Items for Systematic Reviews and Meta-Analyses (PRISMA) criteria to identify studies related to *BRCA1* and *BRCA2* alleles, with a specific focus on Mexican genotypes. Comprehensive searches were performed across the PubMed, SciELO, and EBSCO databases using keywords such as 'Mexican', 'Polymorphism', 'Pathogenic variants', 'BRCA1', 'BRCA2', and other related terms to ensure the inclusion of all relevant articles. Studies published between 2002 and 2023 were included, with the final consultation of sources conducted in September 2023 (Figure 1).

### Inclusion and exclusion criteria

Studies investigating *BRCA1* and *BRCA2* pathogenic variants in Mexican genotypes were included, focusing on patients diagnosed with HBOC as well as individuals without cancer (control group). Articles published in either Spanish or English (the only two languages showing results) were analyzed. The sequencing method or cancer subgroups, such as those based on family cancer history, early onset cancer, or immunohistochemistry markers, were not criteria for discrimination; therefore, all these categories were included in this screening.

Patients who were not Mexican or lacked verifiable Mexican ancestry (i.e., patients from Chile, Brazil, Spain, and others) were not included. Similarly, studies involving human tissues under in vitro, in vivo, or ex vivo conditions, as well as those using non-human subjects or tissues, were also excluded.

### Data selection

**Figure 2:**
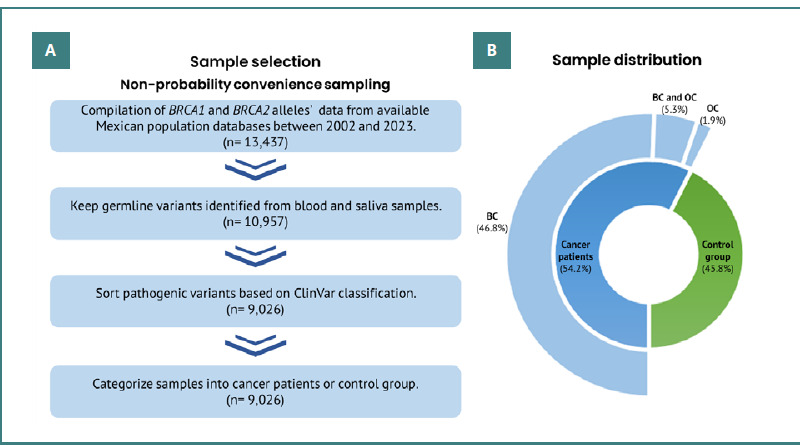
Sample selection and overall incidence in the current study. A, Sample selection was conducted using non-probability convenience sampling, and data were filtered following the depicted steps. B, The pie chart displays the sample distribution after classifying the interest groups.

Two hundred seventy articles were initially retrieved. Eligible articles were evaluated. After reviewing the titles and abstracts of each article, two hundred thirty-two studies were excluded because of the exclusion criteria. After a full review of the remaining 38 studies, another 17 studies were excluded due to the type of sample. All studies that sequenced BRCA genes from tumor tissues were excluded due to the potential inclusion of somatic mutations, which do not reflect germline variations [26]. Additionally, studies reporting polymorphisms that were not reviewed and classified as pathogenic variants by ClinVar were removed. We only retained confirmed pathogenic variants validated by ClinVar (Figure 2A).

### Quality assessment

Standardization of data and pairing nomenclature were performed. Subsequently, the database was enriched by incorporating data from reputable global resources and data-sharing platforms, such as Genome Data Viewer, dbSNP, ClinVar, gnomAD browser, Breast Cancer Information Core (BIC), ClinGen, Varsome, Human Genome Variation Society (HGVS), Bioproject, Ensembl, Gene NIH NCIB, UniProt, and BRCA Exchange.

### Data extraction

While acknowledging the limitations of this investigation, data collection relied on a non-probability convenience sampling approach with limited data from non-cancerous cases. Despite this, a meticulously constructed and comprehensive database containing all reported alleles of *BRCA1* and *BRCA2* in Mexican population studies was developed.

The following data were analyzed: name of the first author, publication date, study design and sample size, cancer diagnosis (and cancer subtype if applicable), region of the study, and all *BRCA1* and *BRCA2* pathogenic variants reported (previously curated using ClinVar classification).

The sample distribution (Figure 2B) was categorized into two primary groups: the control group (45.8%), comprising all alleles not associated with cancer patients (men and women), and the cancer group (54.2%). The cancer group was further subdivided into BC patients (46.8%), OC patients (1.9%), and patients diagnosed with both types of cancers (BC and OC, 5.3%). For the cancer group, inclusion criteria consisted of patients diagnosed with BC, OC, or both types of cancers (no men were found). For the control group, inclusion criteria were less stringent due to the focus on germline variations; individuals of both sexes were included as long as they lacked a personal history of cancer at the time of analysis. The exclusion criterion for the control group was any documented oncological diagnosis.

### Gene map construction

A gene map was constructed to systematically organize all the pathogenic variations identified within the Mexican genotypes. These variations were categorized into two groups: single nucleotide variants (SNVs) and large rearrangements.

## RESULTS

This study performed a comprehensive literature review and data mining to investigate germline pathogenic variation in *BRCA1* and *BRCA2* within the Mexican ancestry. Adhering to the inclusion and exclusion criteria outlined previously, 21 relevant studies were published between 2002 and 2023 (Table 1).

**Table 1:** Characteristics of the studies included in the in-silico analysis of pathogenic alleles of *BRCA1* and *BRCA2* found in the Mexican population

Regions or cities	Cohort size	Group type	Inclusion criteria	Allele count		References
				*BRCA1*	*BRCA2*	
**Guadalajara and Mexico City**	252	BC	Unrelated patients with sporadic BC	25	12	[9]
**Mexico City**	39	BC and OC	BC or OC patients with second or third-degree family history	-	2	[11]
**Monterrey and Mexico City**	188	BC and OC	Patients under 50 years with TNBC and patients with OC with known histological diagnoses	34	-	[25]
**Monterrey and Mexico City**	190	BC	BC patients under 50 years, with TNBC, with a family history of cancer	43	1	[27]
**Mexico City, Monterrey, and Veracruz**	810	BC	BC patients aged 35 to 69 years	20	14	[28]
**Mexico City and Jalisco**	632	BC and OC	BC or OC patients	57	8	[29]
**Coahuila, Nuevo Leon, and Tamaulipas**	195	BC and control group	BC patients over 50 years old, positive for ER and PR	-	3	[30]
**Mexico City**	179	OC	OC patients in clinical stages IA to IVB	35	15	[31]
**Mexico City**	66	BC	BC patients with a family history of cancer	10	15	[32]
**Mexico City**	143	Control group	Data from the Mexican Genome Diversity Project (MGDP)	1	-	[32]
**Mexico City**	3842	Control group	Data from Slim Initiative in Genomics Medicine for the Americas Type 2 Diabetes Whole-Exome Sequence Project (SIGMA Type 2 Diabetes)	4	15	[32]
**Mexico City**	22	BC	Patients with early-onset BC under 35 years old	2	-	[33]
**Mexican population from Southern California**	66	BC and OC	BC and OC patients with a family history of cancer	21	4	[34]
**Population from the southwestern United States with five generations of Mexican ancestry**	1074	BC and OC	BC and OC patients with a family history of cancer	60	19	[35]
**Mexico City, Nuevo Leon, Guadalajara, and Oaxaca**	387	BC	TNBC patients	80	14	[36]
**Hispanics with Mexican ancestry on the border between Texas and Chihuahua (El Paso)**	88	BC	BC patients	11	2	[37]
**State of Mexico and Mexico City**	116	BC and OC	HBOC patients	2	1	[38]
**State of Mexico and Mexico City**	198	BC and OC	Unrelated patients with BC or OC	13	9	[39]
**Monterrey, northeastern region of Mexico**	205	BC, OC, and other exclude cancer types	Cancer patients	25	9	[40]
**Monterrey**	51	BC	Patients with early-onset BC under 35 years old	1	1	[41]
**Monterrey and Mexico City**	53	BC	Patients with invasive BC	4	4	[42]
Mexico City	190	BC	TNBC patients over 50 years	41	1	[43]

A ClinVar search identified 14,798 variations in *BRCA1*, including 4,072 PVs and 19,083 variations in *BRCA2*, of which 5,028 were PVs. Collectively, the studies included in the data mining revealed a total of 657 germline PVs in *BRCA1* and *BRCA2* among the Mexican population. Notably, *BRCA1* appeared to be the more frequently affected gene, with 501 of these PVs attributed to it, while *BRCA2* accounted for the remaining 156. Among these variants, 80 recurrent PVs in *BRCA1* and 59 in *BRCA2* were identified (Table 2).

**Table 2:** Comparison of Mexican *BRCA1* and *BRCA2* PVs in ClinVar in relation to all *BRCA1* and *BRCA2* in this database

Gene	Reported variants	Pathogenic variants	Pathogenic variants in the Mexican population
*BRCA1*	14,798	4,072	80
*BRCA2*	19,083	5,028	59

**Figure 3:**
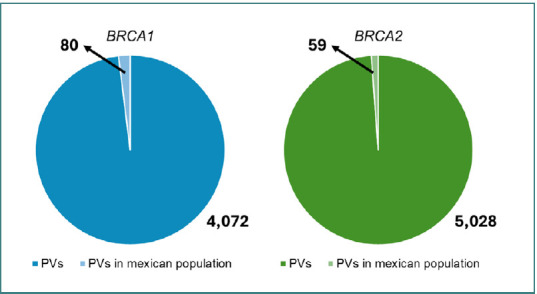
The proportion of ClinVar-Reported PVs for *BRCA1* and *BRCA2* worldwide compared to those in the Mexican population only: 1.96% for *BRCA1* and 1.17% for *BRCA2* exclusive to the Mexican population.

It is important to note the significant difference between the total ClinVar-reported PVs in *BRCA1* and *BRCA2* and those reported specifically in the Mexican population. PVs in the Mexican population accounted for only 1.96% of *BRCA1* and 1.17% of *BRCA2* PVs (Figure 3). This disparity suggests that the Mexican population harbors a distinct set of variations, potentially making it a unique genomic population compared to others. Understanding these specific PVs could be crucial for developing tailored diagnostic and therapeutic strategies.

**Figure 4:**
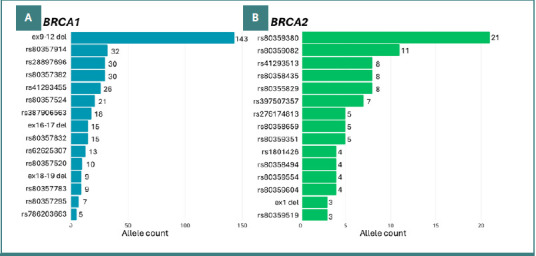
Recurrent PVs of (A) *BRCA1* and (B) *BRCA2*. It only shows the most common fifteen PVs in Mexicans. For the complete data, consult Additional File 1.

As previously mentioned, our data mining revealed 80 recurrent PVs in *BRCA1* and 59 in *BRCA2* within the Mexican population. Figure 4AB summarizes the most common 15 PVs identified. The complete dataset, including detailed study specifications and additional data from other databases, is available in Supplementary File S1.

Supplementary File

**Figure 5:**
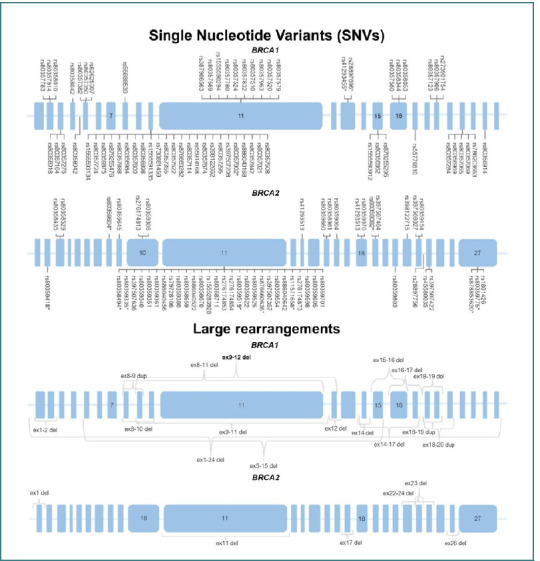
Gene maps of *BRCA1* and *BRCA2* PVs in the Mexican population. The blue boxes represent exons, while the lines represent introns. A) Schematic representation illustrates all SNVs identified in the Mexican population for each gene. Notably, exon 10 in *BRCA1* and exon 11 in *BRCA2* had a higher quantity of SNVs. SNVs in the control group are denoted with an asterisk (*). B) Schematic representation of pathogenic large rearrangements identified in the Mexican population, with *BRCA1* being the most affected gene.

These PVs can be classified into two groups: SNVs and large rearrangements. In both *BRCA1* and *BRCA2* genes, SNVs were the most common type of PVs. Specifically, *BRCA1* exhibited 63 different SNVs and 17 large rearrangements, while *BRCA2* had 53 SNVs and six large rearrangements. Figure 5 illustrates all the PVs identified in the Mexican genotypes in *BRCA1* and *BRCA2*.

Remarkably, only 12 SNVs in the control group were found, but none of the studies reported pathogenic large rearrangements in *BRCA1* and *BRCA2*.

Germline SNVs affecting the Mexican genotypes primarily resulted in deletions, frameshift, stop-gained variants, and missense consequences in the messenger RNA of BRCA genes. Notably, exon 10 in *BRCA1* and exon 11 in *BRCA2* were the most affected by pathogenic SNVs, as evidenced in Figure 5.

Finally, the most frequently observed pathogenic variant in this compilation was the large rearrangement *BRCA1 ex9-12del*, affecting a total of 143 patients across all the studies analyzed. This variant significantly outnumbers the second most common PV, rs80357914, a frameshift SNV in *BRCA1*, with 32 cases.

## DISCUSSION

Genetic diversity among different populations provides valuable insight into understanding the inheritance patterns of PVs in genes associated with complex diseases like cancer. Population studies of mutations in high-penetrance genes, such as *BRCA1* and *BRCA2*, are crucial due to their strong association with breast and ovarian cancer [32]. These studies highlight the importance of sequencing genomes in specific populations, as this allows for the identification of founder mutations: those originating from a single mutational event, are transmitted by a common ancestor, and are frequently found in specific populations. This approach is especially relevant for Hispanic populations, where these genetic factors have not yet been sufficiently explored (Ossa & Torres, 2016)[44]

Despite the extensive study of *BRCA1* and *BRCA2* genes regarding their variation status across various populations, data mining reveals a scarcity of sequencing studies focused on exploring germline mutations in the Mexican population. This lack of research underscores a significant gap in understanding the pathogenic status of *BRCA1* and *BRCA2* in this population.

Although additional studies focused on Mexicans were identified, many did not meet the inclusion criteria established for this compilation. These exclusions were mainly due to incomplete data, lack of review or validation by ClinVar, or the possible presence of somatic variation. The decision to exclude variations of somatic origin was based on the objective of specifically compiling PVS reported in the Mexican population. This could contribute to enriching diagnostic and therapeutic methods by focusing on variations associated with the risk of developing breast or ovarian cancer throughout life, influenced by hereditary and ethnic factors [45].

On the other hand, somatic variations, occurring unpredictably and having limited relevance in early or familial diagnosis, can develop in affected cells before cancer manifests. This can cause genomic aberrations within tumor cells, but their detection would not provide relevant information for genetic predisposition to these diseases [26].

An important limitation of data mining was the use of non-probability convenience sampling, which introduced bias in the stratification of the analyzed population. This was reflected in a similar distribution between the control group and the cancer group, which may not represent the true proportion of patients with breast or ovarian cancer in Mexico in 2019, where 18.5 new cases of breast or ovarian cancer were found per 100,000 inhabitants [46]. Additionally, the composition of the group would not provide a complete view of the status of pathogenic variations in the population unaffected by these diseases.

The data showed that the PVs found in the Mexican population constitute a small percentage of the total variants reported in ClinVar: 1.96% for the *BRCA1* gene and 1.17% for *BRCA2*. This low percentage highlights the genetic uniqueness of the Mexican population. The low frequency of these variants could be related to unique inheritance patterns that distinguish the Mexican population from others, including the possible presence of specific founder variants.

The high prevalence of breast cancer as the leading cause of death from neoplastic diseases among Mexican women underscores the importance of these findings. According to the data mining, 10.26% of Mexican patients with breast or ovarian cancer carry PVs in *BRCA1* and 3.19% in *BRCA2*. This suggests that a significant proportion of these cancers in Mexico may be linked to variations in these genes.

It is relevant to highlight that most PVs found in the Mexican genotypes were detected in the *BRCA1* gene. Additionally, it was observed that the most affected exons were exon 10 for *BRCA1* and exon 11 for *BRCA2*, accumulating the highest number of PVs. These regions are important as they are OCCR and BCCR. Specifically, exon 10 of the *BRCA1* gene is in an OCCR region, while exon 11 of the *BRCA2* gene is part of a BCCR region [20,47].

The identification of these variation accumulation sites is crucial for understanding inheritance patterns and the population behavior of these pathologies and suggests the possibility of considering these exons as new targets for the development of more accessible diagnostic panels for Mexican ancestry.

It is imperative to highlight the importance of large rearrangements in *BRCA1* and *BRCA2*, especially the *BRCA1 ex9-12del* rearrangement, which is the most frequent in the compiled database. This variation has clinical relevance due to its high recurrence rate and prevalence in Mexicans. Additionally, it has been identified as a variation with a founder effect, estimated to have emerged 1480 years ago and exclusively reported in patients with Mexican ancestry [35]. This rearrangement affects approximately 3 KB of the mRNA, including critical protein sites such as the RAD51 binding site and the phosphorylation site, but it must be verified with other studies, such as crystallography (UniProt).

Currently, due to its importance in the region, different strategies have been developed to detect this PV. Traditional methods such as endpoint PCR and multiplex ligation-dependent probe amplification (MLPA) have been used for its identification. However, more recent advances have adapted qPCR to detect this PV in a more sensitive, accessible, and rapid manner [48]. This development underscores the importance of developing new and improved methods for personalized medicine, ensuring that they are accessible and practical for clinical application.

## CONCLUSION

This review and analysis of PVs in the germline of the *BRCA1* and *BRCA2* genes in Mexican ancestry have yielded important findings. A total of 657 pathogenic variants were identified in these genes, with a significant predominance in *BRCA1*. The most affected exons were exon 10 for *BRCA1* and exon 11 for *BRCA2*, which are important due to their association with susceptibility regions for breast and ovarian cancer. Additionally, long rearrangements, especially *BRCA1 ex9-12del*, were found to be recurrent mutations with significant clinical impact in the Mexican genome.

These findings highlight the importance of understanding the genetic profile of the Mexican population in relation to BC and OC, as well as the need to develop more accessible and sensitive diagnostic methods to detect these PVs. Early identification of mutations in *BRCA1* and *BRCA2* can have important implications for the diagnosis, treatment, and management of patients at high genetic risk of developing breast and ovarian cancer.

It is essential to continue researching and improving techniques for detecting and understanding these genetic variants in the Mexican population, especially considering the high incidence of BC and OC in this population. The development of more accurate and accessible diagnostic methods can significantly impact personalized medicine and cancer prevention in Mexico and other countries with similar populations.
