# Supplementary material for: Pathogenic variants in BRCA1 and BRCA2 genes associated with female breast and ovarian cancer in the Mexican population
Source: J Med Life. 2025 Jan;18(1):38–47. doi: 10.25122/jml-2024-0213 (PMC11891617; doi:10.25122/jml-2024-0213)
Supplement: Supplementary file 1 [file JMedLife-18-038-s001.pdf]

Supplementary Material

Table S1. BRCA-1

Main autor	Year	Study reported ID	dbSNP	Transcript identifier	BIC	HGVS RNA	HGVS Protein	Allele ID (ClinVar)	Exon	Locus	Mutation	Molecular consequence	Allele frequency	Allele frequency Latinamerican	Type of mutation
Calderón-Garcia	2005	G2364T	rs80357114	NM_007294.3(BRCA1):c.2245G>T	D749Y	c.2245G>T	p.(Asp749Tyr)	69179	10	17:43093286 (GRCh38.p13)	C>A / C>G / C>T	M			SNV
Calderón-Garcia	2005	3587delT	rs886040168	NM_007294.3(BRCA1):c.3837_3840del	3956del4-ter1159	c.3837_3840del	p.(Ser1280ArgfsTer26)	261705	10	17:43091691-43091694 (GRCh38.p13)	delAGAT	F			Del
Chavarri-Guerra	2022	c.5123C>A	rs28897696	NM_007294.3	5242C>A	c.5123C>A	p.(Ala1708Glu)	70074	13	17:43063903	G>A / G>C / G>T	M	A=0.000091	A=0.000, T=0.000	SNV
Chavarri-Guerra	2022	c.815_824dupAGCCATGTGG	rs387906563	NM_007294.4(BRCA1):c.815_824dup	T108fs	c.815_824dup	p.Thr276fs	70390		17:43094707-43094723 (GRCh38.p14) Help	dupGCTCCACATG	Indel	dupGCTCCACATG=0.000023	CCACATGGCTCCACATGGCTCCACATG=0.000	frameshif
Chavarri-Guerra	2022	c.3037_3038delGA	rs397507208	NM_007294.4(BRCA1):c.3037_3038del (p.Glu1013fs)	3156delGA	c.3037_3038del	p.Glu1013fs	46058		17: 43092493-43092494 (GRCh38)	delCT	D	delCT=0.000004 (1/251124, GnomAD_exome)delCT=0.000008 (1/121256, ExAC)	0	frameshif
Chavarri-Guerra	2022	c.4327C>T	rs41293455	NM_007294.3	4446C>T	c.4327C>T	p.(Arg1443Ter)	17676	13	17:43082434	G>A / G>C	S	0.00010	A=0.000, C=0.000	SNV
Chavarri-Guerra	2022	c.1860delT	rs730881459	NM_007294.4(BRCA1):c.1860del (p.His621fs)	1860delT	c.1860del	p.His621fs	180871		17: 43093671 (GRCh38)	delA	Indel	0	0	frameshif
Chavarri-Guerra	2022	c.5353C>T	rs80356969	NM_007294.3(BRCA1):c.5353C>T	Q1785X	c.5353C>T	p.(Gln1785Ter)	70213	21	17:43049174 (GRCh38.p13)	G>A / G>C / G>T	S			SNV
Chavarri-Guerra	2022	c.5359T>A	rs80357065	NM_007294.3(BRCA1):c.5359T>A	C1787S	c.5359T>A	p.(Cys1787Ser)		21	17:43049168 (GRCh38.p13)	A>C / A>G / A>T	M	T=0.000004	T=0.000	SNV
Chavarri-Guerra	2022	c.5346G>A	rs80357284	NM_007294.4(BRCA1):c.5346G>A	W1782X	c.5346G>A	(p.Trp1782Ter)	46214	21	17: 43049181	C>A / C>G / C>T	S			SNV
Chavarri-Guerra	2022	c.241C>T	rs80357350	NM_007294.4(BRCA1):c.241C>T	241C>T	c.241C>T	p.Gln81Ter	69232		17:43104928 (GRCh38.p14)	G>A / G>C / G>T	Stop	A=0.00006 (1/16830, ALFA)	G=0A=0	SNV
Chavarri-Guerra	2022	c.211A>G	rs80357382	NM_007294.3	R71G	c.211A>G	p.(Arg71Gly)	17693	5	17: 43106457 (on Assembly GRCh38)	T>A / T>C / T>G	M	0.000004	C=0.000	SNV
Chavarri-Guerra	2022	c.211A>G	rs80357382	NM_007294.3	R71G	c.211A>G	p.(Arg71Gly)	17693	5	17: 43106457 (on Assembly GRCh38)	T>A / T>C / T>G	M	0.000004	C=0.000	SNV
Chavarri-Guerra	2022	c.211A>G	rs80357382	NM_007294.3	R71G	c.211A>G	p.(Arg71Gly)	17693	5	17: 43106457 (on Assembly GRCh38)	T>A / T>C / T>G	M	0.000004	C=0.000	SNV
Chavarri-Guerra	2022	c.211A>G	rs80357382	NM_007294.3	R71G	c.211A>G	p.(Arg71Gly)	17693	5	17: 43106457 (on Assembly GRCh38)	T>A / T>C / T>G	M	0.000004	C=0.000	SNV
Chavarri-Guerra	2022	c.211A>G	rs80357382	NM_007294.3	R71G	c.211A>G	p.(Arg71Gly)	17693	5	17: 43106457 (on Assembly GRCh38)	T>A / T>C / T>G	M	0.000004	C=0.000	SNV
Chavarri-Guerra	2022	c.4065_4068del	rs80357508	NM_007294.3(BRCA1):c.4065_4068del	3333del4, 4184 4187delTCAA, 4184del4	c.4065_4068del	p.(Asn1355LysfsTer10)	32713	10	17:43091463-43091468	delGATT	F	delGATT=0.000014	TT=0.000	Indel
Chavarri-Guerra	2022	c.3029_3030delCT	rs80357510	NM_007294.4(BRCA1):c.3029_3030del	3148delCT	c.3029_3030delCT	p.Pro1010Argfs	69424	11	17: 43092501-43092502 (GRCh38)		d	AG=1.00000delAG=0.00000	AG=1.000=0.000	
Chavarri-Guerra	2022	c.3759_3760delTA	rs80357520	NM_007294.3	3878delTA	c.3759_3760del	p.(Lys1254GlufsTer12)	37544	11	17:43091771-43091772	delTA	F			
Chavarri-Guerra	2022	c.1961delA	rs80357522	NM_007294.3(BRCA1):c.1961delA (p.Lys654Serfs)	2080delA	c.1961delA	p.Lys654Serfs	69084		17: 43093569-43093570 (GRCh38)	del(T)4 / delTT / delT / dupT	Indel	delT=0.000008 (2/264690, TOPMED)	TTTTTTTT=1.000TTTT=0.000, TTTTTTT=0.000	frameshif
Chavarri-Guerra	2022	c.2433delC	rs80357524	NM_007294.3	2552delC	c.2433del	p.(Lys812ArgfsTer3)	236075	11	17:43093098-43093101	delG / dupG	F	0,000008	GGG=0.000	
Chavarri-Guerra	2022	c.2433delC	rs80357524	NM_007294.3	2552delC	c.2433del	p.(Lys812ArgfsTer3)	236075	11	17:43093098-43093101	delG / dupG	F	0,000008	GGG=0.000	
Chavarri-Guerra	2022	c.2433delC	rs80357524	NM_007294.3	2552delC	c.2433del	p.(Lys812ArgfsTer3)	236075	11	17:43093098-43093101	delG / dupG	F	0,000008	GGG=0.000	
Chavarri-Guerra	2022	c.2433delC	rs80357524	NM_007294.3	2552delC	c.2433del	p.(Lys812ArgfsTer3)	236075	11	17:43093098-43093101	delG / dupG	F	0,000008	GGG=0.000	
Chavarri-Guerra	2022	c.2433delC	rs80357524	NM_007294.3	2552delC	c.2433del	p.(Lys812ArgfsTer3)	236075	11	17:43093098-43093101	delG / dupG	F	0,000008	GGG=0.000	
Chavarri-Guerra	2022	c.2433delC	rs80357524	NM_007294.3	2552delC	c.2433del	p.(Lys812ArgfsTer3)	236075	11	17:43093098-43093101	delG / dupG	F	0,000008	GGG=0.000	
Chavarri-Guerra	2022	c.2433delC	rs80357524	NM_007294.3	2552delC	c.2433del	p.(Lys812ArgfsTer3)	236075	11	17:43093098-43093101	delG / dupG	F	0,000008	GGG=0.000	
Chavarri-Guerra	2022	c.2433delC	rs80357524	NM_007294.3	2552delC	c.2433del	p.(Lys812ArgfsTer3)	236075	11	17:43093098-43093101	delG / dupG	F	0,000008	GGG=0.000	
Chavarri-Guerra	2022	c.2433delC	rs80357524	NM_007294.3	2552delC	c.2433del	p.(Lys812ArgfsTer3)	236075	11	17:43093098-43093101	delG / dupG	F	0,000008	GGG=0.000	
Chavarri-Guerra	2022	c.5030_5033delCTAA	rs80357580	NM_007294.4(BRCA1):c.5030_5033del (p.Thr1677fs)	5147del4	c.5030_5033del	p.Thr1677fs	46179		17: 43067649-43067652 (GRCh38)	delGTTA / dupGTTA	S	delGTTA=0.000004 (1/264690, TOPMED)delGTTA=0.000007 (1/140172, GnomAD)	TTAGTTA=1.000TTA=0.000	indel
Chavarri-Guerra	2022	c.5030_5033delCTAA	rs80357580	NM_007294.4(BRCA1):c.5030_5033del (p.Thr1677fs)	5147del4	c.5030_5033del	p.Thr1677fs	46179		17: 43067649-43067652 (GRCh38)	delGTTA / dupGTTA	S	delGTTA=0.000004 (1/264690, TOPMED)delGTTA=0.000007 (1/140172, GnomAD)	TTAGTTA=1.000TTA=0.000	indel
Chavarri-Guerra	2022	c.5030_5033delCTAA	rs80357580	NM_007294.4(BRCA1):c.5030_5033del (p.Thr1677fs)	5147del4	c.5030_5033del	p.Thr1677fs	46179		17: 43067649-43067652 (GRCh38)	delGTTA / dupGTTA	S	delGTTA=0.000004 (1/264690, TOPMED)delGTTA=0.000007 (1/140172, GnomAD)	TTAGTTA=1.000TTA=0.000	indel
Chavarri-Guerra	2022	c.1674delA	rs80357600	NM_007294.4(BRCA1):c.1674del (p.Gly559fs)	1793delA	c.1674del	p.Gly559fs	404716		17: 43093856-43093857 (GRCh38)	delTT / delT / dupT	Indel	0	0	frameshif
Chavarri-Guerra	2022	c.2806_2809delGATA	rs80357832	NM_007294.3	2925del4	c.2806_2809del	p.(Asp936SerfsTer63)	37491	11	17:43092722-43092726	delATCT	F	0.000004	T=0.000	
Chavarri-Guerra	2022	c.3858_3861delTGAG	rs80357842	NC_000017.10:g.41243689_41243692del	3975del4, 3977del4	c.3858_3861del	p.(Ser1286ArgfsTer20)	46109	10	17:43091670-43091675	delCACT	F	delCACT=0.000008	CT=0.000	
Chavarri-Guerra	2022	c.3648dupA	rs80357902	NM_007294.4(BRCA1):c.3648dup (p.Ser1217fs)	3767insA	c.3648dup	p.Ser1217fs	46091		17: 43091882-43091883 (GRCh38)	dupT	dup	dupT=0.000004 (1/251312, GnomAD_exome)dupT=0.000008 (1/121296, ExAC)	0	frameshif
Chavarri-Guerra	2022	c.5266dupC	rs80357906	NM_007294.3	5382 5383insC, 5382insC	c.5266dup	p.(Gln1756ProfsTer74)	32716	19	17:43057063-43057065	dupG	F	0.000034	GGGG=0.000	Indel
Chavarri-Guerra	2022	c.66_67delAG	rs80357914	NM_007294.3	185 186delAG, 185delAB, 185delAG	c.68_69del	p.(Glu23ValfsTer17)	32701	2	17:43124028-43124029 (GRCh38)	delAG	F	0.000227	CT=0.000, CTCTCT=0.000	
Chavarri-Guerra	2022	c.80+1G>A	rs80358010	NM_007294.4(BRCA1):c.80+1G>A	IVS2+1G>A	c.-109+1G>A		131055		17: 43124016 (GRCh38)	C>A / C>G / C>T				SNV
Chavarri-Guerra	2022	c.81-1G>A	rs80358018	NM_007294.3(BRCA1):c.81-1G>C				855796		17:43115780	C>A / C>G / C>T			T=0.000004 (1/250180, GnomAD_exome) T=0.000009 (1/109242, ExAC) T=0.000 (0/478, ALFA)	
Chavarri-Guerra	2022	c.5074+1G>A	rs80358053	NM_007294.3(BRCA1):c.5074+1G>A	IVS17+1G>A	c.5074+1G>C		262943		17: 41219624 (GRCh37)	C>A / C>G / C>T	SDV	T=0.000004 (1/251352, GnomAD_exome)	0	SNV
Chavarri-Guerra	2022	c.5062_5064delGTT	rs80358344	NM_007294.3(BRCA1):c.5062_5064delGTT (p.Val1688del)	V1688del	c.5062_5064delGTT	p.Val1688del	70035		17: 43067618-43067620 (GRCh38)	delACA / dupACA	inframe del	0	0	indel
Chavarri-Guerra	2022	c.1407_1408delAA	rs879255476	NM_007294.4(BRCA1):c.1407_1408del (p.Ser470fs)	1526delAA	c.1407_1408del	p.Ser470fs	247264		17: 43094123-43094124 (GRCh38)	delTT	D	0	0	frameshif
Chavarri-Guerra	2022	ex1-2del			del exon 1-2		del exon 1-2								
Chavarri-Guerra	2022	ex1-24del			del exon 1-24		del exon 1-24								
Chavarri-Guerra	2022	ex14del			del exon 14-14		del exon 14								
Chavarri-Guerra	2022	ex14-17dup			del exon 14-17		del exon 14-17								
Chavarri-Guerra	2022	ex16-17del		NM_007294.3(BRCA1):c.4676-?_5074+?del	del exon 16-17		del exon 16-17	213237	16-17	17: 43067608-43071238		D			
Chavarri-Guerra	2022	ex16-17del		NM_007294.3(BRCA1):c.4676-?_5074+?del	del exon 16-17		del exon 16-17	213237	16-17	17: 43067608-43071238		D			
Chavarri-Guerra	2022	ex16-17del		NM_007294.3(BRCA1):c.4676-?_5074+?del	del exon 16-17		del exon 16-17	213237	16-17	17: 43067608-43071238		D			

[illegible]

Supplementary Material

Table S2. BRCA-2

Main autor	Year	Study reported ID	dbSNP	Transcript identifier	BIC	HGVS RNA	HGVS Protein	Allele ID (ClinVar)	Exon	Ñocus	Mutation	Molecular consequence	Allele frequency	Allele frequency Latinamerican	Type of mutation
Calderón-García	2005	A10462T	rs1801426	NM_000059.3(BRCA2):c.10234A>G	10462 A>G	c.10234A>G	p.(Ile3412Val)	49979	27	13:32398747 (GRCh38.p13)	A>G	M	G=0.044743	G=0.0787	SNV
Calderón-García	2005	A10462T	rs1801426	NM_000059.3(BRCA2):c.10234A>G	10462 A>G	c.10234A>G	p.(Ile3412Val)	49979	27	13:32398747 (GRCh38.p13)	A>G	M	G=0.044743	G=0.0787	SNV
Calderón-García	2005	A10462T	rs1801426	NM_000059.3(BRCA2):c.10234A>G	10462 A>G	c.10234A>G	p.(Ile3412Val)	49979	27	13:32398747 (GRCh38.p13)	A>G	M	G=0.044743	G=0.0787	SNV
Calderón-García	2005	A10462T	rs1801426	NM_000059.3(BRCA2):c.10234A>G	10462 A>G	c.10234A>G	p.(Ile3412Val)	49979	27	13:32398747 (GRCh38.p13)	A>G	M	G=0.044743	G=0.0787	SNV
Calderón-García	2005	G8605A	rs80359082	NM_000059.3(BRCA2):c.8377G>A	G2793R	c.8377G>A	p.(Gly2793Arg)	249055	19	13:32370447 (GRCh38.p13)	G>A / G>T	S	A=0.000008	A=0.000	SNV
Calderón-García	2005	A3199G	rs886040456	NM_000059.3(BRCA2):c.2971_2983del	3199del13	c.2971_2983del	p.(Asn991AspfsTer3)	261165	11	13:32337325-32337338 (GRCh38.p13)	delAACAAATGGGCAG	F			Indel
Calderón-García	2005	A3199G	rs886040456	NM_000059.3(BRCA2):c.2971_2983del	3199del13	c.2971_2983del	p.(Asn991AspfsTer3)	261165	11	13:32337325-32337338 (GRCh38.p13)	delAACAAATGGGCAG	F			Indel
Chavarri-Guerra	2022	c.5631delC	rs397507357	NM_000059.3(BRCA2):c.5631del	N1877Kfs*32	c.5631del	p.(Asn1877LysfsTer32)	46537	11	13:32339986 (GRCh38.p13)	delC	F	delC=0.000008		D
Chavarri-Guerra	2022	c.8168A>G	rs41293513	NM_000059.3(BRCA2):c.8168A>G	8396A>G	c.8168A>G	p.(Asp2723Gly)	46697	18	13:32363370 (GRCh38.p13)	A>C / A>G / A>T	M	G=0.000004	G=0.000	SNV
Chavarri-Guerra	2022	c.274C>T	rs80358529	NM_000059.4(BRCA2):c.274C>T (p.Gln92Ter)	502C>T	c.274C>T	(p.Gln92Ter)	46355		13: 32319283 (GRCh38)	C>A / C>G / C>T	S	T=0.00000 (0/78696, PAGE_STUDY)T=0.000 (0/660, ALFA)	C=0T=0	SNV
Chavarri-Guerra	2022	c.274C>T	rs80358529	NM_000059.4(BRCA2):c.274C>T (p.Gln92Ter)	502C>T	c.274C>T	(p.Gln92Ter)	46355		13: 32319283 (GRCh38)	C>A / C>G / C>T	S	T=0.00000 (0/78696, PAGE_STUDY)T=0.000 (0/660, ALFA)	C=0T=0	SNV
Chavarri-Guerra	2022	c.274C>T	rs80358529	NM_000059.4(BRCA2):c.274C>T (p.Gln92Ter)	502C>T	c.274C>T	(p.Gln92Ter)	46355		13: 32319283 (GRCh38)	C>A / C>G / C>T	S	T=0.00000 (0/78696, PAGE_STUDY)T=0.000 (0/660, ALFA)	C=0T=0	SNV
Chavarri-Guerra	2022	c.4111C>T	rs80358659	NM_000059.3(BRCA2):c.4111C>T	Q1371X	c.4111C>T	p.(Gln1371Ter)	66267	11	13:32338466 (GRCh38.p13)	C4339T	S	T=0.00003	T=0	SNV
Chavarri-Guerra	2022	c.4889C>G	rs80358711	NM_000059.4(BRCA2):c.4889C>G (p.Ser1630Ter)	5117C>G	c.4889C>G	(p.Ser1630Ter)	66400		13: 32339244 (GRCh38)	C>A / C>G / C>T	s	G=0.000008 (2/248994, GnomAD_exome)G=0.000007 (1/140088, GnomAD)G=0.000017 (2/119522, ExAC)	0	SNV
Chavarri-Guerra	2022	c.2653_2656delGACA	rs80359340	NM_000059.4(BRCA2):c.2653_2656del (p.Asp885fs)	2881del4	c.2653_2656del	(p.Asp885fs)	65990	11	13: 32337006-32337009 (GRCh38)	delGACA	F	delGACA=0.000009 (2/228720, GnomAD_exome)delGACA=0.000017 (2/120120, ExAC)	0	indel
Chavarri-Guerra	2022	c.2808_2811delACAA	rs80359351	NM_000059.3(BRCA2):c.2808_2811del	3034del4, 3036 3039delACAA, 3036del4, 3036delACAA	c.2808_2811del	p.(Ala938ProfsTer21)	24361	11	13:32337161-32337166	delACAA	F	delACAA=0.000023	AA=0.000	Indel
Chavarri-Guerra	2022	c.3264dupT	rs80359380	NM_000059.3(BRCA2):c.3264dup	3492insT	c.3264dup	p.(Gln1089SerfsTer10)	46386	11	13:32337619 (GRCh38.p13)	dupT / insTT	F	dupT=0.000026	TT=0.000	Indel
Chavarri-Guerra	2022	c.6024dupG	rs80359554	NM_000059.3(BRCA2):c.6024dupG (p.Gln2009Alafs)	6252insG	c.6024dupG	(p.Gln2009Alafs)	46571	11	13: 32340378-32340379 (GRCh38)	dupG	F	dupG=0.000004 (1/264690, TOPMED)dupG=0.000004 (1/250690, GnomAD_exome)dupG=0.00000 (0/14050, ALFA)	G=1.000GG=0.000	Indel
Chavarri-Guerra	2022	c.658_659delGT	rs80359604	NM_000059.3(BRCA2):c.658_659del	886delGT	c.658_659delGT	p.(Val220IlefsTer4)	24381	8	13:32329468-32329470 (GRCh38.p13)	delGT	F	delGT=0.000053	T=0.000	Indel
Chavarri-Guerra	2022	c.6591_6592delTG	rs80359605	NM_000059.3(BRCA2):c.6591_6592delTG (p.Glu2198Asnfs)	6819delTG	c.6591_6592delTG	(p.Glu2198Asnfs)	24358	11	13: 32340946-32340947 (GRCh38)	delTG	F	delTG=0.000004 (1/264690, TOPMED)delTG=0.000004 (1/246418, GnomAD_exome)delTG=0.00000 (0/10680, ALFA)	TG=1.000=0.000	Del
Chavarri-Guerra	2022	c.9699_9702delTATG	rs80359775	NM_000059.3:c.9699_9702delTATG	9927del4	c.9699_9702del	p.(Cys3233TrpfsTer15)	46816	27	13:32398210-32398215 (GRCh38.p13)	delTATG	F	delTATG=0.000064	TG=0.000	Indel
Fernández López	2019	rs11571658	rs11571658	NM_000059.3(BRCA2):c.6275_6276del	6503delTT	c.6275_6276delTT	p.Leu2092Profs	24357	11	13:32340630-32340631 (GRCh38.p13)	delTT	F	delTT=0.000037	0	del
Fernández López	2019	9463delG	rs397507422	NM_000059.3(BRCA2):c.9235del	V3079Ffs*4	c.9235del	p.(Val3079PhefsTer4)	46777	24	13:32380124 (GRCh38.p13)	delG	F	delG=0.000076	0	del
Fernández López	2019	rs41293513	rs41293513	NM_000059.3(BRCA2):c.8168A>G	8396A>G	c.8168A>G	p.(Asp2723Gly)	46697	18	13:32363370 (GRCh38.p13)	A>C / A>G / A>T	M	G=0.000004	G=0.000	SNV
Fernández López	2019	rs80358494	rs80358494	NM_000059.3(BRCA2):c.2224C>T	p.Gln742*	c.2224C>T	p.(Gln742Ter)	65925	11	13:32336579	C>A / C>G / C>T	S	T=0.000008	A=0.000, T=0.000	SNV
Fernández López	2019	rs80359082	rs80359082	NM_000059.3(BRCA2):c.8377G>A	G2793R	c.8377G>A	p.(Gly2793Arg)	249055	19	13:32370447 (GRCh38.p13)	G>A / G>T	S	A=0.000008	A=0.000	SNV
Fernández López	2019	rs80359082	rs80359082	NM_000059.3(BRCA2):c.8377G>A	G2793R	c.8377G>A	p.(Gly2793Arg)	249055	19	13:32370447 (GRCh38.p13)	G>A / G>T	S	A=0.000008	A=0.000	SNV
Fernández López	2019	rs80359082	rs80359082	NM_000059.3(BRCA2):c.8377G>A	G2793R	c.8377G>A	p.(Gly2793Arg)	249055	19	13:32370447 (GRCh38.p13)	G>A / G>T	S	A=0.000008	A=0.000	SNV
Fernández López	2019	Rs80359335	rs80359335	NM_000059.3(BRCA2):c.2588dup	2810insA, 2816insA	c.2588dup	p.(Asn863LysfsTer18)	999923	11	13:32336937-32336943 (GRCh38.p13)	delA / dupA	F	dupA=0.000009		Indel
Fernández López	2019	2900delCT	rs80359361	NM_000059.3(BRCA2):c.2899_2900del	3127delCT	c.2899_2900del	p.(Leu967ArgfsTer14)	131536	11	13:32337252-32337255 (GRCh38.p13)	delCT	F			Indel
Fernández López	2019	rs777107618 rs80359380	rs80359380	NM_000059.3(BRCA2):c.3264dup	3492insT	c.3264dup	p.(Gln1089SerfsTer10)	46386	11	13:32337619 (GRCh38.p13)	dupT / insTT	F	dupT=0.000026	TT=0.000	Indel
Fernández López	2019	rs80359418	rs80359418	NM_000059.3(BRCA2):c.3del	231delG	c.3_3delG	p.Met1Ilefs	46428	2	13:32316463 (GRCh38.p13)	delG	F			del
Fernández López	2019	rs80359519	rs80359519	NM_000059.3(BRCA2):c.5542del	5770delA	c.5542del	p.(Ser1848ValfsTer15)	66546	11	13:32339897 (GRCh38.p13)	delA	F	delA=0.000008		del
Fernández López	2019	6252insG	rs80359554	NM_000059.3(BRCA2):c.6024dup	6252insG	c.6024dup	p.(Gln2009AlafsTer9)	46571	11	13:32340379 (GRCh38.p13)	dupG	F	dupG=0.000004	GG=0.000	Indel
Fernández López	2019	6714del4	rs80359598	NM_000059.3(BRCA2):c.6486_6489del	6714 6717delACAA, 6714del4	c.6486_6489del	p.(Lys2162AsnfsTer5)	46604	11	13:32340837-32340844	delACAA	F	delACAA=0.000023	ACAA=0.000	Indel
Fernández López	2019	rs80359604	rs80359604	NM_000059.3(BRCA2):c.658_659del	886delGT	c.658_659delGT	p.(Val220IlefsTer4)	24381	8	13:32329468-32329470 (GRCh38.p13)	delGT	F	delGT=0.000053	T=0.000	Indel
Fernández López	2019	rs80359660	rs80359660	NM_000059.3(BRCA2):c.7556dup	7784insC	c.7556dup	p.(Arg2520SerfsTer19)	96959	15	13:32356547-32356548 (GRCh38.p13)	dupC	F	dupC=0.000004		Indel
Fernández López	2019	rs80359775	rs80359775	NM_000059.3:c.9699_9702delTATG	9927del4	c.9699_9702del	p.(Cys3233TrpfsTer15)	46816	27	13:32398210-32398215 (GRCh38.p13)	delTATG	F	delTATG=0.000064	TG=0.000	Indel
Fernández López	2019	Rs876660636	rs876660636	NM_000059.4(BRCA2):c.658_659del	886 887delGT, 886delGT	c.658_659del	p.(Val220IlefsTer4)	24381	11	13:32339982-32339985 (GRCh38.p13)	delA	F			Indel
Fernández López	2019	Rs878853620	rs878853620	NM_000059.3:c.9686C>A		c.9686C>A	p.(Pro3229His)	641907	27	13:32398199 (GRCh38.p13)	C>A / C>T	F			SNV
Fernández López	2019	6244delG	rs886040642	NM_000059.3(BRCA2):c.6244G>T	6472G>T, p.Glu2082X	c.6244G>T	p.(Glu2082Ter)	261344	11	13:32340599 (GRCh38.p13)	G>T	S			SNV
Fernández López	2019	Ex1del			del exon1										
Fernández López	2019	Ex23del			del exon23										
Fernández López	2019	Ex1del			del exon1										
Fernández López	2019	Ex17del			del exon17										
Fernández López	2019	Ex23del			del exon23										
Fernández López	2019	Ex22-24del			del exon22-24										
Fernández López	2019	Ex22-24del			del exon22-24										
Fernández López	2019	Ex11del			del exon11										
Fernández López	2019	Ex26del			del exon26										
Fernández López	2019	Ex1del			del exon1										
Gallardo Rincón	2020	c.4749_4750delAG	rs1555283928	NM_000059.3(BRCA2):c.4749del	I1583Mfs*34	c.4749del	p.(Ile1583MetfsTer34)	432799	11	13:32339103-32339104 (GRCh38.p13)	delT	F			Indel
Gallardo Rincón	2020	c.1796e1800 del CTTAT	rs276174813	NM_000059.3(BRCA2):c.1796_1800del	2022del5, 2024 2028delCTTAT, 2024del5, p.Ser599*	c.1796_1800del	p.(Ser599Ter)	46312	10	13:32333272-32333278 (GRCh38.p13)	delCTTAT	S	delCTTAT=0.000004		Indel
Gallardo Rincón	2020	c.1796e1800 del CTTAT	rs276174813	NM_000059.3(BRCA2):c.1796_1800del	2022del5, 2024 2028delCTTAT, 2024del5, p.Ser599*	c.1796_1800del	p.(Ser599Ter)	46312	10	13:32333272-32333278 (GRCh38.p13)	delCTTAT	S	delCTTAT=0.000004		Indel
Gallardo Rincón	2020	c.5116_5119 del AATA	rs276174853	NM_000059.3(BRCA2):c.5116_5119del	5344delAATA	c.5116_5119del	p.(Asn1706LeufsTer5)	66441	11	13:32339467-32339474 (GRCh38.p13)	delAATA	F	delAATA=0.000007		Indel
Gallardo Rincón	2020	c.5116e5119 del AATA	rs276174853	NM_000059.3(BRCA2):c.5116_5119del	5344delAATA	c.5116_5119del	p.(Asn1706LeufsTer5)	66441	11	13:32339467-32339474 (GRCh38.p13)	delAATA	F	delAATA=0.000007		Indel
Gallardo Rincón	2020	c.5631delC	rs397507357	NM_000059.3(BRCA2):c.5631del	N1877Kfs*32	c.5631del	p.(Asn1877LysfsTer32)	46537	11	13:32339986 (GRCh38.p13)	delC	F	delC=0.000008		del
Gallardo Rincón	2020	c.8168 A > G	rs41293513	NM_000059.3(BRCA2):c.8168A>G	8396A>G	c.8168A>G	p.(Asp2723Gly)	46697	18	13:32363370 (GRCh38.p13)	A>C / A>G / A>T	M	G=0.000004	G=0.000	SNV
Gallardo Rincón	2020	c.8168 A > G	rs41293513	NM_000059.3(BRCA2):c.8168A>G	8396A>G	c.8168A>G	p.(Asp2723Gly)	46697	18	13:32363370 (GRCh38.p13)	A>C / A>G / A>T	M	G=0.000004	G=0.000	SNV
Gallardo Rincón	2020	c.8168 A > G	rs41293513	NM_000059.3(BRCA2):c.8168A>G	8396A>G	c.8168A>G	p.(Asp2723Gly)	46697	18	13:32363370 (GRCh38.p13)	A>C / A>G / A>T	M	G=0.000004	G=0.000	SNV
Gallardo Rincón	2020	c.8168 A > G	rs41293513	NM_000059.3(BRCA2):c.8168A>G	8396A>G	c.8168A>G	p.(Asp2723Gly)	46697	18	13:32363370 (GRCh38.p13)	A>C / A>G / A>T	M	G=0.000004	G=0.000	SNV
Gallardo Rincón	2020	c.8168 A > G	rs41293513	NM_000059.3(BRCA2):c.8168A>G	8396A>G	c.8168A>G	p.(Asp2723Gly)	46697	18	13:32363370 (GRCh38.p13)	A>C / A>G / A>T	M	G=0.000004	G=0.000	SNV
Gallardo Rincón	2020	c.4325 C > A	rs80358670	NM_000059.3(BRCA2):c.4325C>A	S1442X	c.4325C>A	p.(Ser1442Ter)	97297	11	13:32338680 (GRCh38.p13)	C>A / C>G / C>T	S	A=0.000004	A=0	SNV
Gallardo Rincón	2020	c.4325 C > A	rs80358670	NM_000059.3(BRCA2):c.4325C>A	S1442X	c.4325C>A	p.(Ser1442Ter)	97297	11	13:32338680 (GRCh38.p13)	C>A / C>G / C>T	S	A=0.000004	A=0	SNV
Gallardo Rincón	2020	c.5542 del A	rs80359519	NM_000059.3(BRCA2):c.5542del	5770delA	c.5542del	p.(Ser1848ValfsTer15)	66546	11	13:32339897 (GRCh38.p13)	delA	F	delA=0.000008		del
Gallardo Rincón	2020	c.5616_5620delAGTAA	rs80359525	NM_000059.3(BRCA2):c.5616_5620del	5844del5	c.5616_5620del	p.(Lys1872AsnfsTer2)	66560	11	13:32339966-32339975 (GRCh38.p13)	delAGTAA	F	delAGTAA=0.000049	AGTAA=0.000	Indel
Herzog, Chavarri	2021	c.5631del (p.Asn1877fs)	rs397507357	NM_000059.3(BRCA2):c.5631del	N1877Kfs*32	c.5631del	p.(Asn1877LysfsTer32)	46537	11	13:32339986 (GRCh38.p13)	delC	F	delC=0.000008		D
Herzog, Chavarri	2021	c.5631del (p.Asn1877fs)	rs397507357	NM_000059.3(BRCA2):c.5631del	N1877Kfs*32	c.5631del	p.(Asn1877LysfsTer32)	46537	11	13:32339986 (GRCh38.p13)	delC	F	delC=0.000008		D
Herzog, Chavarri	2021	c.5631del (p.Asn1877fs)	rs397507357	NM_000059.3(BRCA2):c.5631del	N1877Kfs*32	c.5631del	p.(Asn1877LysfsTer32)	46537	11	13:32339986 (GRCh38.p13)	delC	F	delC=0.000008		D
Herzog, Chavarri	2021	c.2808_2811del (p.Ala938Profs)	rs80359351	NM_000059.3(BRCA2):c.2808_2811del	3034del4, 3036 3039delACAA, 3036del4, 3036delACAA	c.2808_2811del	p.(Ala938ProfsTer21)	24361	11	13:32337161-32337166	delACAA	F	delACAA=0.000023	AA=0.000	Indel
Herzog, Chavarri	2021	c.2808_2811del (p.Ala938Profs)	rs80359351	NM_000059.3(BRCA2):c.2808_2811del	3034del4, 3036 3039delACAA, 3036del4, 3036delACAA	c.2808_2811del	p.(Ala938ProfsTer21)	24361	11	13:32337161-32337166	delACAA	F	delACAA=0.000023	AA=0.000	Indel
Herzog, Chavarri	2021	c.3264dupT (p.Gln1089Serfs)	rs80359380	NM_000059.3(BRCA2):c.3264dup	3492insT	c.3264dup	p.(Gln1089SerfsTer10)	46386	11	13:32337619 (GRCh38.p13)	dupT / insTT	F	dupT=0.000026	TT=0.000	Indel
Herzog, Chavarri	2021	c.3264dupT (p.Gln1089Serfs)	rs80359380	NM_000059.3(BRCA2):c.3264dup	3492insT	c.3264dup	p.(Gln1089SerfsTer10)	46386	11	13:32337619 (GRCh38.p13)	dupT / insTT	F	dupT=0.000026	TT=0.000	Indel
Herzog, Chavarri	2021	c.3264dupT (p.Gln1089Serfs)	rs80359380	NM_000059.3(BRCA2):c.3264dup	3492insT	c.3264dup	p.(Gln1089SerfsTer10)	46386	11	13:32337619 (GRCh38.p13)	dupT / insTT	F	dupT=0.000026	TT=0.000	Indel
Millan Catalán	2019	c.5631delC	rs397507357	NM_000059.3(BRCA2):c.5631del	N1877Kfs*32	c.5631del	p.(Asn1877LysfsTer32)	46537	11	13:32339986 (GRCh38.p13)	delC	F	delC=0.000008		D
Millan Catalán	2019	c.3166C>T	rs79728106	NM_000059.3(BRCA2):c.3166C>T	Q1056X	c.3166C>T	p.(Gln1056Ter)	66080	11	13:32337521 (GRCh38.p13)	C>A / C>G / C>T	S	T=0.00001	T=0.000	SNV
Millan Catalán	2019	c.145G>T	rs80358435	NM_000059.3(BRCA2):c.145G>T	p.Glu49*	c.145G>T	p.(Glu49Ter)	65797	3	13:32319154 (GRCh38.p13) Help	G>C / G>T	S	T=0.000	T=0	SNV
Millan Catalán	2019	c.8219T>G	rs80359070	NM_000059.3(BRCA2):c.8219T>A	L2740X	c.8219T>A	p.(Leu2740Ter)	24382	18	13:32363421 (GRCh38.p13)	T>A	S	A=0.00000		SNV
Millan Catalán	2019	c.1806insA	rs80359306	NM_000059.3(BRCA2):c.1813del	2034delA, 2041 2042delA, 2041delA	c.1813del	p.(Ile605TyrfsTer9)	46318	10	13:32333284-32333291 (GRCh38.p13)	delA / dupA	F	dupA=0.000029	AAAAAAAAA=0.000	Indel
Millan Catalán	2019	c.1813delA	rs80359306	NM_000059.3(BRCA2):c.1813del	2034delA, 2041 2042delA, 2041delA	c.1813del	p.(Ile605TyrfsTer9)	46318	10	13:32333284-32333291 (GRCh38.p13)	delA / dupA	F	dupA=0.000029	AAAAAAAAA=0.000	Indel
Millan Catalán	2019	c.2899_2900delCT	rs80359361	NM_000059.3(BRCA2):c.2899_2900del	3127delCT	c.2899_2900del	p.(Leu967ArgfsTer14)	131536	11	13:32337252-32337255 (GRCh38.p13)	delCT	F			Indel
Millan Catalán	2019	c.3492insT	rs80359380	NM_000059.3(BRCA2):c.3264dup	3492insT	c.3264dup	p.(Gln1089SerfsTer10)	46386	11	13:32337619 (GRCh38.p13)	dupT / insTT	F	dupT=0.000026	TT=0.000	Indel
Millan Catalán	2019	c.6024_6025insG	rs80359554	NM_000059.3(BRCA2):c.6024dup	6252insG	c.6024dup	p.(Gln2009AlafsTer9)	46571	11	13:32340379 (GRCh38.p13)	dupG	F	dupG=0.000004	GG=0.000	Indel
Millan Catalán	2019	c.6486_6489delACAA	rs80359598	NM_000059.3(BRCA2):c.6486_6489del	6714 6717delACAA, 6714del4	c.6486_6489del	p.(Lys2162AsnfsTer5)	46604	11	13:32340837-32340844	delACAA	F	delACAA=0.000023	ACAA=0.000	Indel
Millan Catalán	2019	c.8754G>A	rs80359803	NM_000059.3(BRCA2):c.8754G>A	8982G>A	c.8754G>A	p.(Glu2918=)	67339	21	13:32376791 (GRCh38.p13)	G>A	Sy	A=0.000004	A=0	SNV
Millan Catalán	2019	c.6244_6244delG	rs886040642	NM_000059.3(BRCA2):c.6244G>T	6472G>T, p.Glu2082X	c.6244G>T	p.(Glu2082Ter)	261344	11	13:32340599 (GRCh38.p13)	G>T	S			SNV
Nahleh	2015	E49X (373G>T)	rs80358435	NM_000059.3(BRCA2):c.145G>T	p.Glu49*	c.145G>T	p.(Glu49Ter)	65797	3	13:32319154 (GRCh38.p13) Help	G>C / G>T	S	T=0.000	T=0	SNV
Nahleh	2015	3492insT	rs80359380	NM_000059.3(BRCA2):c.3264dup	3492insT	c.3264dup	p.(Gln1089SerfsTer10)	46386	11	13:32337619 (GRCh38.p13)	dupT / insTT	F	dupT=0.000026	TT=0.000	Indel
Oliver	2019	c.G7007A	rs28897743	NM_000059.4(BRCA2):c.7007G>A (p.Arg2336His)		c.7007G>A	(p.Arg2336His)	66909		13: 32346896 (GRCh38)	G>A / G>C / G>T	M	A=0.000011 (3/264690, TOPMED)C=0.000004 (1/247222, GnomAD_exome)A=0.000080 (10/124316, ALFA)	G=1.000A=0.000, C=0.000	SNV
Ossa Gomez	2022	NM_000059.3:c.8839G>T	rs398122715	NM_000059.4(BRCA2):c.8839G>T (p.Glu2947Ter)	9067G>T	c.8839G>T	(p.Glu2947Ter)	97213		13: 32379401 (GRCh38)	G>A / G>C / G>T	S	T=0.00001 (1/78578, PAGE_STUDY)T=0.000 (0/658, ALFA)	G=0T=0	SNV
Ossa Gomez	2022	NM_000059.3:c.8168A>G	rs41293513	NM_000059.3(BRCA2):c.8168A>G	8396A>G	c.8168A>G	p.(Asp2723Gly)	46697	18	13:32363370 (GRCh38.p13)	A>C / A>G / A>T	M	G=0.000004	G=0.000	SNV
Ossa Gomez	2022	NM_000059.3:c.145G>T	rs80358435	NM_000059.3(BRCA2):c.145G>T	p.Glu49*	c.145G>T	p.(Glu49Ter)	65797	3	13:32319154 (GRCh38.p13) Help	G>C / G>T	S	T=0.000	T=0	SNV
Ossa Gomez	2022	NM_000059.3:c.4111C>T	rs80358659	NM_000059.3(BRCA2):c.4111C>T	Q1371X	c.4111C>T	p.(Gln1371Ter)	66267	11	13:32338466 (GRCh38.p13)	C4339T	S	T=0.00003	T=0	SNV
Ossa Gomez	2022	NM_000059.3:c.9018C>A	rs80359154	NM_000059.4(BRCA2):c.9018C>A (p.Tyr3006Ter)	9018C>A	c.9018C>A	(p.Tyr3006Ter)	484916		13: 32379814 (GRCh38)	C>A / C>G / C>T	S	A=0.00003 (2/78698, PAGE_STUDY)A=0.000 (0/658, ALFA)	C=0A=0	SNV
Ossa Gomez	2022	NM_000059.3:c.2808_2811delACAA	rs80359351	NM_000059.3(BRCA2):c.2808_2811del	3034del4, 3036 3039delACAA, 3036del4, 3036delACAA	c.2808_2811del	p.(Ala938ProfsTer21)	24361	11	13:32337161-32337166	delACAA	F	delACAA=0.000023	AA=0.000	Indel
Ossa Gomez	2022	NM_000059.3:c.3264dupT	rs80359380	NM_000059.3(BRCA2):c.3264dup	3492insT	c.3264dup	p.(Gln1089SerfsTer10)	46386	11	13:32337619 (GRCh38.p13)	dupT / insTT	F	dupT=0.000026	TT=0.000	Indel
Ossa Gomez	2022	NM_000059.3:c.3264dupT	rs80359380	NM_000059.3(BRCA2):c.3264dup	3492insT	c.3264dup	p.(Gln1089SerfsTer10)	46386	11	13:32337619 (GRCh38.p13)	dupT / insTT	F	dupT=0.000026	TT=0.000	Indel
Ossa Gomez	2022	NM_000059.3:c.658_659delGT	rs80359604	NM_000059.3(BRCA2):c.658_659del	886delGT	c.658_659delGT	p.(Val220IlefsTer4)	24381	8	13:32329468-32329470 (GRCh38.p13)	delGT	F	delGT=0.000053	T=0.000	Indel
Pérez-Ibave	2023	BRCA2 c.9154C>T (p.Arg3052Trp)	rs45580035	NM_000059.4(BRCA2):c.9154C>T (p.Arg3052Trp)	9382C>T	c.9154C>T	(p.Arg3052Trp)	67431		13: 32380043 (GRCh38)	C>A / C>G / C>T	M	T=0.000012 (3/251190, GnomAD_exome)T=0.000008 (1/120724, ExAC)T=0.00007 (6/89514, ALFA)	C=1.000T=0.000	SNV
Pérez-Ibave	2023	c.274C>T (p.Gln92*)	rs80358529	NM_000059.4(BRCA2):c.274C>T (p.Gln92Ter)	502C>T	c.274C>T	(p.Gln92Ter)	46355		13: 32319283 (GRCh38)	C>A / C>G / C>T	S	T=0.00000 (0/78696, PAGE_STUDY)T=0.000 (0/660, ALFA)	C=0T=0	SNV
Pérez-Ibave	2023	c.274C>T (p.Gln92Ter)	rs80358529	NM_000059.4(BRCA2):c.274C>T (p.Gln92Ter)	502C>T	c.274C>T	(p.Gln92Ter)	46355		13: 32319283 (GRCh38)	C>A / C>G / C>T	S	T=0.00000 (0/78696, PAGE_STUDY)T=0.000 (0/660, ALFA)	C=0T=0	SNV
Pérez-Ibave	2023	c.274C>T (p.Gln92Ter)	rs80358529	NM_000059.4(BRCA2):c.274C>T (p.Gln92Ter)	502C>T	c.274C>T	(p.Gln92Ter)	46355		13: 32319283 (GRCh38)	C>A / C>G / C>T	S	T=0.00000 (0/78696, PAGE_STUDY)T=0.000 (0/660, ALFA)	C=0T=0	SNV
Pérez-Ibave	2023	c.274C>T (p.Gln92Ter)	rs80358529	NM_000059.4(BRCA2):c.274C>T (p.Gln92Ter)	502C>T	c.274C>T	(p.Gln92Ter)	46355		13: 32319283 (GRCh38)	C>A / C>G / C>T	S	T=0.00000 (0/78696, PAGE_STUDY)T=0.000 (0/660, ALFA)	C=0T=0	SNV
Pérez-Ibave	2023	BRCA2 c.274C>T (p.Gln92*)	rs80358529	NM_000059.4(BRCA2):c.274C>T (p.Gln92Ter)	502C>T	c.274C>T	(p.Gln92Ter)	46355		13: 32319283 (GRCh38)	C>A / C>G / C>T	S	T=0.00000 (0/78696, PAGE_STUDY)T=0.000 (0/660, ALFA)	C=0T=0	SNV
Pérez-Ibave	2023	c.7558C>T (p.Arg2520*)	rs80358981	NM_000059.4(BRCA2):c.7558C>T (p.Arg2520Ter)	R2520*	c.7558C>T	(p.Arg2520Ter)	67021		13: 32356550 (GRCh38)	C>A / C>G / C>T	S	T=0.000019 (5/264690, TOPMED)T=0.000020 (5/251188, GnomAD_exome)T=0.000018 (3/166362, ALFA)	C=1.000T=0.000	SNV
Pérez-Ibave	2023	c.7558C>T (p.Arg2520*)	rs80358981	NM_000059.4(BRCA2):c.7558C>T (p.Arg2520Ter)	R2520*	c.7558C>T	(p.Arg2520Ter)	67021		13: 32356550 (GRCh38)	C>A / C>G / C>T	S	T=0.000019 (5/264690, TOPMED)T=0.000020 (5/251188, GnomAD_exome)T=0.000018 (3/166362, ALFA)	C=1.000T=0.000	SNV
Pérez-Ibave	2023	c.8377G>A (p.Gly2793Arg)	rs80359082	NM_000059.3(BRCA2):c.8377G>A	G2793R	c.8377G>A	p.(Gly2793Arg)	249055	19	13:32370447 (GRCh38.p13)	G>A / G>T	S	A=0.000008	A=0.000	SNV
Ruiz-Flores	2002	8605G>A	rs80359082	NM_000059.4(BRCA2):c.8377G>A (p.Gly2793Arg)	8605G>A	c.8377G>A	p.(Gly2793Arg)	249055		13: 32370447 (GRCh38)	G>A / G>C / G>T	S	A=0.000004 (1/264690, TOPMED)A=0.000008 (2/251416, GnomAD_exome)A=0.000008 (1/121406, ExAC)	G=1.000A=0.000	SNV
Torres-Mejía	2015	c.1796-1800delTTTAT	rs276174813	NM_000059.3(BRCA2):c.1796_1800del	2022del5, 2024 2028delCTTAT, 2024del5, p.Ser599*	c.1796_1800del	p.(Ser599Ter)	46312	10	13:32333272-32333278 (GRCh38.p13)	delCTTAT	S	delCTTAT=0.000004		Indel
Torres-Mejía	2015	c.1796-1800delTTTAT	rs276174813	NM_000059.3(BRCA2):c.1796_1800del	2022del5, 2024 2028delCTTAT, 2024del5, p.Ser599*	c.1796_1800del	p.(Ser599Ter)	46312	10	13:32333272-32333278 (GRCh38.p13)	delCTTAT	S	delCTTAT=0.000004		Indel
Torres-Mejía	2015	c.1796-1800delTTTAT	rs276174813	NM_000059.3(BRCA2):c.1796_1800del	2022del5, 2024 2028delCTTAT, 2024del5, p.Ser599*	c.1796_1800del	p.(Ser599Ter)	46312	10	13:32333272-32333278 (GRCh38.p13)	delCTTAT	S	delCTTAT=0.000004		Indel
Torres-Mejía	2015	P2153L	rs276174873	NM_000059.3(BRCA2):c.6458C>T	P2153L	c.6458C>T	p.(Pro2153Leu)	66777	11	13:32340813 (GRCh38.p13)	C>T	M	T=0.000009	T=0.000	SNV
Torres-Mejía	2015	5859delC	rs397507357	NM_000059.3(BRCA2):c.5631del	N1877Kfs*32	c.5631del	p.(Asn1877LysfsTer32)	46537	11	13:32339986 (GRCh38.p13)	delC	F	delC=0.000008		D
Torres-Mejía	2015	c.4111C>T	rs80358659	NM_000059.3(BRCA2):c.4111C>T	Q1371X	c.4111C>T	p.(Gln1371Ter)	66267	11	13:32338466 (GRCh38.p13)	C4339T	S	T=0.00003	T=0	SNV
Torres-Mejía	2015	c.4111C>T	rs80358659	NM_000059.3(BRCA2):c.4111C>T	Q1371X	c.4111C>T	p.(Gln1371Ter)	66267	11	13:32338466 (GRCh38.p13)	C4339T	S	T=0.00003	T=0	SNV
Torres-Mejía	2015	c.4111C>T	rs80358659	NM_000059.3(BRCA2):c.4111C>T	Q1371X	c.4111C>T	p.(Gln1371Ter)	66267	11	13:32338466 (GRCh38.p13)	C4339T	S	T=0.00003	T=0	SNV
Torres-Mejía	2015	c.2808-2811delACAA	rs80359351	NM_000059.3(BRCA2):c.2808_2811del	3034del4, 3036 3039delACAA, 3036del4, 3036delACAA	c.2808_2811del	p.(Ala938ProfsTer21)	24361	11	13:32337161-32337166	delACAA	F	delACAA=0.000023	AA=0.000	Indel
Torres-Mejía	2015	c.3264-3265insT	rs80359380	NM_000059.3(BRCA2):c.3264_3265insTT	Q1089Ffs*16	c.3264_3265insTT	p.(Gln1089PhefsTer16)	66104	11	13:32337619 (GRCh38.p13)	dupT / insTT	F	dupT=0.000023	TT=0.000	Indel
Torres-Mejía	2015	c.5542delA	rs80359519	NM_000059.3(BRCA2):c.5542del	5770delA	c.5542del	p.(Ser1848ValfsTer15)	66546	11	13:32339897 (GRCh38.p13)	delA	F	delA=0.000008		D
Torres-Mejía	2015	c.6486-6489delACAA	rs80359598	NM_000059.3(BRCA2):c.6486_6489del	6714 6717delACAA, 6714del4	c.6486_6489del	p.(Lys2162AsnfsTer5)	46604	11	13:32340837-32340844	delACAA	F	delACAA=0.000023	ACAA=0.000	Indel
Torres-Mejía	2015	2971del5	rs80359701	NM_000059.3(BRCA2):c.8247_8248del	8474delAG, 8475 8476delGA, 8475delGA	c.8247_8248del	p.(Lys2750AspfsTer13)	67204	11	13:32363448-32363450 (GRCh38.p13)	delGA	F			Indel
Torres-Mejía	2015	4534delAT	rs886040522	NM_000059.4(BRCA2):c.4306_4307del	4534 4535delAT, p.Ile1436X	c.4306_4307del	p.(Ile1436Ter)	261235	11	13:32338659-32338662 (GRCh38.p13)	delAT	S			Indel
Vaca Paniagua	2012	c.2639_2640delTG	rs397507636	NM_000059.3(BRCA2):c.2637_2638del		c.2637_2638del	p.(Glu880ArgfsTer7)	65987	11	13:32336992-32336993 (GRCh38.p13)	delTG	F			del
Vaca Paniagua	2012	c.5114_5117delTAAA	rs80359522	NM_000059.3(BRCA2):c.5577_5580del	5805del4	c.5577_5580del	p.(Lys1861Ter)	66550	11	13:32339932-32339935	delTAAA	F			del
Vazquez-Juárez	2021	rs276174854	rs276174854	NM_000059.4(BRCA2):c.5146_5149del (p.Tyr1716fs)	5373delGTAT	c.5146_5149del	(p.Tyr1716fs)	66447	11	13: 32339499-32339502 (GRCh38)	delTATG	F	delTATG=0.000004 (1/226616, GnomAD_exome)delTATG=0.000008 (1/118220, ExAC)delTATG=0.0002 (1/6404, 1000(	0	INDEL
Vazquez-Juárez	2021	rs397508027	rs397508027	NM_000059.4(BRCA2):c.8988_8990delinsTT (p.Leu2996fs)	L2996fs	c.8988_8990delinsTT	(p.Leu2996fs)	67389		13: 32379784-32379786 (GRCh38)	delATAinsTT	F	0	0	SNV
Vazquez-Juárez	2021	rs80359554	rs80359554	NM_000059.3(BRCA2):c.6024dup	6252insG	c.6024dup	p.(Gln2009AlafsTer9)	46571	11	13:32340379 (GRCh38.p13)	dupG	F	dupG=0.000004	GG=0.000	Indel
Vazquez-Juárez	2021	rs80359604	rs80359604	NM_000059.3(BRCA2):c.658_659del	886delGT	c.658_659delGT	p.(Val220IlefsTer4)	24381	8	13:32329468-32329470 (GRCh38.p13)	delGT	F	delGT=0.000053	T=0.000	Indel
Villarreal-Garza	2015	2452C[T (Q742X)	rs80358494	NM_000059.3(BRCA2):c.2224C>T	p.Gln742*	c.2224C>T	p.(Gln742Ter)	65925	11	13:32336579	C>A / C>G / C>T	S	T=0.000008	A=0.000, T=0.000	SNV
Villarreal-Garza Influence of Germline BRCA	2021	2452C > T (Q742X)	rs80358494	NM_000059.4(BRCA2):c.2224C>T (p.Gln742Ter)	2452C>T	c.2224C>T	(p.Gln742Ter)	65925		13: 32336579 (GRCh38)	C>A / C>G / C>T	S	T=0.000008 (1/121156, ExAC)T=0.00004 (3/78702, PAGE_STUDY)A=0.00000 (0/31068, ALFA)	C=1.000A=0.000, T=0.000	SNV
Weitzel	2005	Q742X	rs80358494	NM_000059.3(BRCA2):c.2224C>T	p.Gln742*	c.2224C>T	p.(Gln742Ter)	65925	11	13:32336579	C>A / C>G / C>T	S	T=0.000008	A=0.000, T=0.000	SNV
Weitzel	2005	W2586X	rs80359004	NM_000059.3(BRCA2):c.7758G>A	W2586X	c.7758G>A	p.(Trp2586Ter)	46672	16	13:32357882 (GRCh38.p13)	G>A / G>C	S	A=0.00001	A=0	SNV
Weitzel	2005	3492insT	rs80359380	NM_000059.3(BRCA2):c.3264dup	3492insT	c.3264dup	p.(Gln1089SerfsTer10)	46386	11	13:32337619 (GRCh38.p13)	dupT / insTT	F	dupT=0.000026	TT=0.000	Indel
Weitzel	2005	957del4	rs80359645	NM_000059.3(BRCA2):c.729_732del	957del4	c.729_732del	p.(Asn243LysfsTer7)	66970	9	13:32330966-32330969 (GRCh38.p13)	delTGAT	F			del
Weitzel, Clague	2013	c.145G¬T	rs80358435	NM_000059.3(BRCA2):c.145G>T	p.Glu49*	c.145G>T	p.(Glu49Ter)	65797	3	13:32319154 (GRCh38.p13) Help	G>C / G>T	S	T=0.000	T=0	SNV
Weitzel, Clague	2013	c.145G¬T	rs80358435	NM_000059.3(BRCA2):c.145G>T	p.Glu49*	c.145G>T	p.(Glu49Ter)	65797	3	13:32319154 (GRCh38.p13) Help	G>C / G>T	S	T=0.000	T=0	SNV
Weitzel, Clague	2013	c.145G¬T	rs80358435	NM_000059.3(BRCA2):c.145G>T	p.Glu49*	c.145G>T	p.(Glu49Ter)	65797	3	13:32319154 (GRCh38.p13) Help	G>C / G>T	S	T=0.000	T=0	SNV
Weitzel, Clague	2013	c.145G¬T	rs80358435	NM_000059.3(BRCA2):c.145G>T	p.Glu49*	c.145G>T	p.(Glu49Ter)	65797	3	13:32319154 (GRCh38.p13) Help	G>C / G>T	S	T=0.000	T=0	SNV
Weitzel, Clague	2013	c.145G¬T	rs80358435	NM_000059.3(BRCA2):c.145G>T	p.Glu49*	c.145G>T	p.(Glu49Ter)	65797	3	13:32319154 (GRCh38.p13) Help	G>C / G>T	S	T=0.000	T=0	SNV
Weitzel, Clague	2013	c.8377G¬A	rs80359082	NM_000059.3(BRCA2):c.8377G>A	G2793R	c.8377G>A	p.(Gly2793Arg)	249055	19	13:32370447 (GRCh38.p13)	G>A / G>T	S	A=0.000008	A=0.000	SNV
Weitzel, Clague	2013	c.8377G¬A	rs80359082	NM_000059.3(BRCA2):c.8377G>A	G2793R	c.8377G>A	p.(Gly2793Arg)	249055	19	13:32370447 (GRCh38.p13)	G>A / G>T	S	A=0.000008	A=0.000	SNV
Weitzel, Clague	2013	c.8377G¬A	rs80359082	NM_000059.3(BRCA2):c.8377G>A	G2793R	c.8377G>A	p.(Gly2793Arg)	249055	19	13:32370447 (GRCh38.p13)	G>A / G>T	S	A=0.000008	A=0.000	SNV
Weitzel, Clague	2013	c.8377G¬A	rs80359082	NM_000059.3(BRCA2):c.8377G>A	G2793R	c.8377G>A	p.(Gly2793Arg)	249055	19	13:32370447 (GRCh38.p13)	G>A / G>T	S	A=0.000008	A=0.000	SNV
Weitzel, Clague	2013	c.3264dupT	rs80359380	NM_000059.3(BRCA2):c.3264dup	3492insT	c.3264dup	p.(Gln1089SerfsTer10)	46386	11	13:32337619 (GRCh38.p13)	dupT / insTT	F	dupT=0.000026	TT=0.000	Indel
Weitzel, Clague	2013	c.3264dupT	rs80359380	NM_000059.3(BRCA2):c.3264dup	3492insT	c.3264dup	p.(Gln1089SerfsTer10)	46386	11	13:32337619 (GRCh38.p13)	dupT / insTT	F	dupT=0.000026	TT=0.000	Indel
Weitzel, Clague	2013	c.3264dupT	rs80359380	NM_000059.3(BRCA2):c.3264dup	3492insT	c.3264dup	p.(Gln1089SerfsTer10)	46386	11	13:32337619 (GRCh38.p13)	dupT / insTT	F	dupT=0.000026	TT=0.000	Indel
Weitzel, Clague	2013	c.3264dupT	rs80359380	NM_000059.3(BRCA2):c.3264dup	3492insT	c.3264dup	p.(Gln1089SerfsTer10)	46386	11	13:32337619 (GRCh38.p13)	dupT / insTT	F	dupT=0.000026	TT=0.000	Indel
Weitzel, Clague	2013	c.3264dupT	rs80359380	NM_000059.3(BRCA2):c.3264dup	3492insT	c.3264dup	p.(Gln1089SerfsTer10)	46386	11	13:32337619 (GRCh38.p13)	dupT / insTT	F	dupT=0.000026	TT=0.000	Indel
Weitzel, Clague	2013	c.3264dupT	rs80359380	NM_000059.3(BRCA2):c.3264dup	3492insT	c.3264dup	p.(Gln1089SerfsTer10)	46386	11	13:32337619 (GRCh38.p13)	dupT / insTT	F	dupT=0.000026	TT=0.000	Indel
Weitzel, Clague	2013	c.3264dupT	rs80359380	NM_000059.3(BRCA2):c.3264dup	3492insT	c.3264dup	p.(Gln1089SerfsTer10)	46386	11	13:32337619 (GRCh38.p13)	dupT / insTT	F	dupT=0.000026	TT=0.000	Indel
Weitzel, Clague	2013	c.3264dupT	rs80359380	NM_000059.3(BRCA2):c.3264dup	3492insT	c.3264dup	p.(Gln1089SerfsTer10)	46386	11	13:32337619 (GRCh38.p13)	dupT / insTT	F	dupT=0.000026	TT=0.000	Indel
Weitzel, Clague	2013	c.3264dupT	rs80359380	NM_000059.3(BRCA2):c.3264dup	3492insT	c.3264dup	p.(Gln1089SerfsTer10)	46386	11	13:32337619 (GRCh38.p13)	dupT / insTT	F	dupT=0.000026	TT=0.000	Indel
Weitzel, Clague	2013	c.3264dupT	rs80359380	NM_000059.3(BRCA2):c.3264dup	3492insT	c.3264dup	p.(Gln1089SerfsTer10)	46386	11	13:32337619 (GRCh38.p13)	dupT / insTT	F	dupT=0.000026	TT=0.000	Indel
Zayas Villanueva	2019	c.9117G>A	rs28897756	NM_000059.3(BRCA2):c.9117G>A	9345G>A	c.9117G>A	p.(Pro3039=)	46771	Intron 23	13:32379913 (GRCh38.p13)	G>A / G>C / G>T	Sy	A=0.000004	A=0.000	SNV
Zayas Villanueva	2019	c.8488-1G>A	rs397507404	NM_000059.3(BRCA2):c.8488-1G>A	IVS19-1G>A	c.8488-1G>A		46720	19	13:32370955 (GRCh38.p13)	G>A	SAV	A=0.000008	A=0.000	SNV
Zayas Villanueva	2019	c.8377G>A	rs80359082	NM_000059.3(BRCA2):c.8377G>A	G2793R	c.8377G>A	p.(Gly2793Arg)	249055	19	13:32370447 (GRCh38.p13)	G>A / G>T	S	A=0.000008	A=0.000	SNV
